# Hypersensitivity reactions to biologics used in the treatment of allergic diseases: clinical features, diagnosis and management

**DOI:** 10.3389/falgy.2023.1219735

**Published:** 2023-08-11

**Authors:** Andrea Sitek, Sergio E. Chiarella, Thanai Pongdee

**Affiliations:** ^1^ Division of Allergy, Clinical Immunology and Rheumatology, University of Kansas Medical Center, Kansas, KS, United States; ^2^ Division of Allergic Diseases, Mayo Clinic, Rochester, MN, United States

**Keywords:** hypersensitivity, anaphylaxis, biologic, monoclonal antibody, desensitization, serum sickness

## Abstract

Several monoclonal antibodies have been approved by the Food and Drug Administration (FDA) to treat allergic disorders, including omalizumab, dupilumab, mepolizumab, reslizumab, benralizumab, tralokinumab and tezepelumab, and their indications continue to expand. Although the risks associated with these agents are overall low, hypersensitivity reactions have been described and are reported more frequently with increased use. We provide a comprehensive review of clinical features, diagnosis and management of hypersensitivity reactions attributed to these agents. We aim to provide useful information for the clinician managing hypersensitivity reactions to these monoclonal antibodies, as well as highlight the need for future research to address specific gaps in knowledge.

## Introduction

Biologic agents have demonstrated efficacy in treating several allergic diseases, and the indications for monoclonal antibodies continue to expand. Several monoclonal antibodies have been approved by the Food and Drug Administration (FDA) to treat allergic disorders including asthma, chronic spontaneous urticaria, chronic rhinosinusitis with nasal polyps, hypereosinophilic syndrome, atopic dermatitis, and eosinophilic esophagitis (Table 1).

**Table 1:** FDA-Approved indications, dosing, reported delayed hypersensitivity reactions and black box warnings of biologics used in allergic disorders.

Biologic	FDA-approved indications	Dosing and route	Delayed hypersensitivity reactions	Black box warnings
Omalizumab	CSU refractory to H1 antihistamine, ≥12 years	CSU: 150 or 300 SC every 4 weeks	Serum sickness and SSLR: 4 cases reported in clinical trials (1), case reports (2, 3)	Anaphylaxis in 0.2% (4)
	IgE-mediated allergic asthma, not controlled by ICS, ≥6 years	Asthma: dose determined by serum IgE level, ranging from 150 to 375 mg SC every 2–4 weeks		
	CRSwNP, not controlled by inhaled corticosteroid, ≥18 years	CRSwNP: dose determined by serum IgE level, ranging from 75 to 600 mg SC every 2–4 weeks		
Mepolizumab	EGPA, ≥18 years	EGPA: 300 mg SC every 4 weeks	None	None
	HES, ≥12 years	HES: 300 mg SC every 4 weeks		
	CRSwNP, inadequate response to nasal corticosteroid, ≥18 years	Nasal polyps: 100 mg SC every 4 weeks		
	Severe eosinophilic asthma, ≥6 years	Asthma: 100 mg SC every 4 weeks for (≥12 years); 40 mg SC every 4 weeks (6–11 years)		
Reslizumab	Severe eosinophilic asthma, ≥18 years	3 mg/kg IV infusion every 4 weeks	None	Anaphylaxis in 0.3% (5)
Benralizumab	Severe eosinophilic asthma, ≥12 years	30 mg SC every 4 weeks for the first 3 doses, then 30 mg every 8 weeks	None	None
Dupilumab	Moderate to severe eosinophilic or steroid-dependent asthma, ≥6 years	Asthma, ≥18 years: 400–600 mg SC loading dose, followed by 200–300 mg SC every 2 weeks	Serum sickness and SSLR: case reports (6, 7)	None
	Moderate to severe AD, not controlled with topical therapies, ≥6 months	Asthma, pediatric: dose determined by age and weight, ranging from 100 to 600 mg SC, followed by 100–300 mg every 2–4 weeks	Erythema multiform: case report (8)	
	CRSwNP, ≥18 years	AD, ≥18 years: 600 mg SC, followed by 300 mg SC every 2 weeks		
	EoE, ≥12 years	AD, pediatric: dose determined by age and weight, ranging from 200 to 600 mg SC, followed by 200–300 mg every 2–4 weeks		
		CRSwNP: 300 mg SC every 2 weeks		
		EoE: 300 mg SC every week		
Tezepelumab	Severe asthma, ≥12 years	210 mg SC every 4 weeks	None	None
Tralokinumab	Moderate to severe AD not controlled by topical therapies, ≥18 years	600 mg SC loading dose, followed by 300 mg SC every 2 weeks	None	None

CSU, chronic spontaneous urticaria; ICS, inhaled corticosteroid; EGPA, eosinophilic granulomatosis with polyangiitis; HES, hypereosinophilic syndrome; CRSwNP, chronic rhinosinusitis with nasal polyposis; EoE, eosinophilic esophagitis; AD, atopic dermatitis; SC, subcutaneous; IV, intravenous; SSLR, serum sickness like reaction.

Although the risks associated with these agents are overall low, hypersensitivity reactions have been described and reported more frequently with increased use (9). Hypersensitivity reactions to monoclonal antibodies may be classified as immediate and delayed. Immediate hypersensitivity reactions include infusion-related reactions, cytokine release reactions, type I (IgE and non-IgE) reactions and mixed reactions (IgE and cytokine-release). Delayed reactions include type III (serum sickness reactions) and type IV reactions (9). Appropriate identification of a hypersensitivity reaction is important to avoid both under- and overdiagnosis.

We provide a comprehensive review of the types of hypersensitivity reactions ascribed to monoclonal antibodies utilized in the management of allergic diseases, including omalizumab, dupilumab, mepolizumab, reslizumab, benralizumab, tralokinumab and tezepelumab (10–15). Hypersensitivity reactions attributed to these agents include type I, type III and type IV reactions (9). Beyond clinical history, skin testing is the most readily available tool to aid in diagnosis of type I hypersensitivity reaction to biologics (9). For patients with suspected immediate-onset allergic reaction, desensitization may offer the ability to continue beneficial treatments (9, 16). We describe currently available data regarding skin testing and desensitization and highlight gaps in knowledge. The information provided in this review should aid in both diagnosing and managing hypersensitivity reactions to these biologic agents.

## Immediate hypersensitivity reactions

Immediate reactions to biologics can be classified as infusion-related reactions, cytokine release reactions and type I (IgE/non-IgE) reactions (9, 17). Anaphylaxis, a type I reaction, has been reported to omalizumab, mepolizumab, dupilumab, reslizumab and benralizumab. Cytokine release and infusion-related reactions have not been attributed to biologics used to treat atopic disease (15). Symptoms of anaphylaxis typically occur within 30–120 min of administration (18). Multiple systems can be involved, including cutaneous, respiratory, gastrointestinal, cardiovascular, and neurologic, and the severity can range from mild urticarial rash to life-threatening anaphylaxis (9). Typically, IgE-mediated reactions need several exposures before occurring, though they can occur after the first dose.

Omalizumab and reslizumab product labels feature black box warnings for anaphylaxis (4, 15). The overall rate of anaphylaxis caused by omalizumab, reslizumab and other biologics utilized for allergic diseases is low, occurring in approximately 0%−0.3% of patients (19). The overall risk of omalizumab-related anaphylaxis is approximately 0.1%−0.2% (19). According to a review of reported data from a post marketing case-control study, 51% of reported cases of anaphylaxis developed after the first dose, and most reactions (77%) occurred within the first three doses (20). Most patients (69%) developed symptoms within 60 min of medication administration; however, many patients experienced delayed anaphylaxis (20). The most commonly reported symptoms were respiratory (96%), and the majority of patients experienced both respiratory and cutaneous symptoms (69%) (20).

In a pooled analysis of clinical trials data, anaphylaxis to reslizumab was identified in four patients (0.3%) (5). One patient was treated with epinephrine; the others were treated with antihistamines and corticosteroids. None experienced respiratory failure, circulatory collapse, or death (5). There are fewer details available about these reactions when compared to omalizumab; the timing of symptom onset and the number of doses received prior to the reaction are not described. An observation period is recommended after the administration of both agents. The American Academy of Allergy, Asthma and Immunology/American College of Allergy, Asthma and Immunology Joint Task Force initially recommended that all patients be observed after each injection, for 2 h after the first 3 injections and 30 min after all subsequent injections (19). For patients with no prior history of anaphylaxis, including to drugs, foods, etc., the FDA allows for omalizumab to be self-administered at home if the patient receives the initial 3 doses under observation with no hypersensitivity reaction. For reslizumab, guidance regarding monitoring is less specific. The package insert recommends observation after reslizumab administration for “an appropriate period of time” (15).

The risk of anaphylaxis with other monoclonal antibodies used to treat allergic disease is very low. The majority of hypersensitivity reactions attributed to benralizumab were mild (e.g., urticarial rash) and occurred at an overall rate of 1%−3% of patients in placebo-controlled trials (21–23). Anaphylaxis was reported in one patient receiving benralizumab in an open-label extension trial (24). Among patients receiving dupilumab in clinical trials, hypersensitivity reactions were reported in approximately 1%, with no difference between placebo and dupilumab (25, 26). Dupilumab's package insert states that hypersensitivity reactions occur in less than 1% of patients, and multiple types of hypersensitivity reaction are listed, including, generalized urticaria, serum sickness, rash, erythema nodosum and anaphylaxis (14). No patients randomized to receive tezepelumab or tralokinumab as part of phase II or phase III clinical trials reported anaphylaxis (27–30). The package inserts for both agents warn about a possible risk of hypersensitivity reaction, including anaphylaxis and angioedema (11, 12).

Most patients who develop anaphylaxis to omalizumab are female (84%), though this association has not been described with other biologic agents (20). Another risk for anaphylaxis to omalizumab includes a prior history of anaphylaxis to other agents, including foods and other medications [odds ratio of 8.1 (95% CI, 2.7–24.3)] (20). Risk factors for developing anaphylaxis to biologics are otherwise not well-defined, and there is no reliable biomarker to identify patients at risk for anaphylaxis to these agents. The immunogenicity of biologics, which is the capability of these agents to stimulate the formation of anti-drug antibodies (ADAs), may contribute to the development of hypersensitivity reactions, and the presence of ADAs has been explored as a potential indicator of risk for hypersensitivity reaction to a few biologics (9). An association between high ADAs of IgE isotype to infliximab and cetuximab and severe hypersensitivity reaction to these agents has been described (31). The relationship between ADA formation and risk of anaphylaxis is not known for omalizumab, reslizumab or other monoclonal antibodies used to treat allergic diseases.

The pathogenesis of anaphylaxis to biologics is incompletely understood. ADAs of both IgG and IgE isotypes are thought to play a role. IgG ADAs may trigger anaphylaxis via complement activation and subsequent release of anaphylatoxins (9). However, the delayed nature of reactions to omalizumab, and the potential for first-dose reactions, suggest that this is not likely the only mechanism (32). Non-humanized biologics carry a higher risk of hypersensitivity reaction than humanized monoclonal antibodies, and it has been proposed that IgE-mediated reactions to omalizumab may be related to ADA formation in response to murine components of the drug (17, 32). Anaphylaxis due to excipient allergy has also been suggested, and a case report describes onset of anaphylaxis to omalizumab thought to be a result of exposure to excipient polysorbate (16, 32). This report describes two patients who developed anaphylaxis after receiving omalizumab for more than 1 year. Skin prick tests to omalizumab were negative, and intradermal skin tests were negative in 1 patient. Intradermal polysorbate testing was positive in this same individual (33).

## Delayed hypersensitivity reactions

Delayed hypersensitivity reactions to biologics can be classified as type III (serum sickness reactions) and type IV hypersensitivity reactions (9). Serum sickness reactions (SSRs) and serum sickness like reactions (SSLRs) have been reported to both omalizumab and dupilumab. Symptoms of SSRs/SSLRs typically occur 5–7 days after drug exposure and can include fever, malaise, myalgia, arthralgia/arthritis, rash, pruritis, edema and purpura (18).

In omalizumab pre-marketing clinical trials, there were four reported incidents of SSLRs. Three cases developed in patients receiving omalizumab and one in the control group (1). Signs and symptoms included arthritis, rash, fever, and lymphadenopathy with an onset 1–5 days after the first or subsequent injections of omalizumab. All cases resolved despite the continuation of treatment. The first case of serum sickness reaction leading to drug discontinuation was described in 2007 in a 67-year-old woman receiving omalizumab 300 mg for severe persistent asthma (34). Symptoms of arthralgia and swelling of the left wrist and lower extremities, malaise and generalized pruritis developed on day 5 post injection. She was found to have left wrist and ankle arthritis, pitting edema of the lower extremities and tenosynovitis of the left-hand extensors. Tenosynovitis was confirmed by MRI, and C reactive protein (CRP) was increased (2.7 mg/dl). Circulating immune complexes were not increased, and complement levels were normal. Symptoms self-resolved; however, they recurred 6 days after an additional dose of omalizumab 300 mg. Patient was treated with 1 g methylprednisolone with resolution of joint and tendon pain, but she developed necrosis of soft tissue over the tendons of the right-hand. Omalizumab was discontinued. The patient ultimately died 40 days after the second omalizumab injection, though this was thought to be related to underlying coronary artery disease (34). Since this time, a few additional case reports have been published, including a case of a 12-year-old patient who developed serum sickness-like reaction after receiving omalizumab for chronic urticaria (2, 3).

Two case reports describe serum sickness reactions to dupilumab (6, 7). In one case, a patient developed type I hypersensitivity reaction on initial exposure to dupilumab, then developed serum sickness reaction with subsequent exposure. This patient developed urticaria and exacerbation of asthma symptoms after their first dupilumab injection, and dupilumab was discontinued (7). Two years later, they underwent dupilumab challenge without immediate reaction; however, 24 h after the challenge dose, they developed symptoms of myalgia, facial and hand swelling, arthralgia, and rash at the injection site. They were found to have elevated inflammatory markers, and serum sickness reaction was suspected. The patient discontinued dupilumab and was treated with prednisone with resolution of symptoms in 3 days (7). In another case, a patient developed SSLR while receiving dupilumab for atopic dermatitis (6).

The pathogenesis of serum sickness reactions is not completely understood, but it is thought to be related to complement-fixing IgM and IgG antibodies formed to biologic-related antigens (9). This results in immune complex deposition in small blood vessels of the skin, kidney, and other organs. Inflammatory markers can be elevated, though this is not specific (34). If clinically significant type III hypersensitivity reactions occur, the culprit agent should be discontinued (16). A challenge could be considered in mild SSLRs with shared decision making, particularly if the diagnosis is uncertain (16). However, no tools are available to aid in risk stratification in such cases.

Type IV hypersensitivity reactions have been attributed to monoclonal antibodies utilized for allergic diseases in a few rare cases. One case report describes erythema multiforme in a patient receiving dupilumab (8). A general warning of “rash” is listed on the package insert of several agents, including tralokinumab, reslizumab and others; however, the type of rash is not specified (12, 15). Maculopapular rash, which is generally a much more common drug reaction than serum sickness reaction, has not been attributed to biologics included in this review. Severe cutaneous adverse events, like Steven's Johnson syndrome and toxic epidermal necrolysis, have also not been reported. Though injection site reactions are among the most common side effects of biologics administered subcutaneously, the frequency of type IV hypersensitivity reactions at the injection site versus other types of injection site reactions is unclear (35).

## Skin testing for biologics

Skin testing can be performed in patients with a suspected type I (IgE-mediated) hypersensitivity reaction to a monoclonal antibody. Most of the available literature regarding skin testing for monoclonal antibodies focuses on TNF-α inhibitors, such as infliximab, adalimumab, and etanercept (9). In contrast, there is a relative lack of studies addressing skin testing for monoclonal antibodies used to treat allergic diseases.

In general, skin testing should be performed at least 4–6 weeks after the reaction to the monoclonal antibody to minimize the likelihood of a false negative result (36). In addition, guidelines recommend starting with an undiluted monoclonal antibody for skin prick testing. If the prick testing is negative, proceed with intradermal testing with 0.03 ml of a 1:100 dilution of the monoclonal antibodies followed by a 1:10 dilution (36). A positive result is a wheal at least 3 mm larger than the negative control. Medications and protocols to manage allergic reactions should be in place when performing skin testing for monoclonal antibodies (9). Notably, the skin testing results can help decide whether to proceed with a graded dose challenge versus a desensitization procedure. Furthermore, it can help with risk stratification before a desensitization procedure (37).

As mentioned, few studies have explored skin testing for monoclonal antibodies used for allergic conditions (7, 38, 39). Lieberman and colleagues reported the results of skin testing for omalizumab in healthy volunteers and patients with allergic asthma, which provided several clinically-relevant insights (38). First, the authors recommend diluting omalizumab in normal saline. Second, they report that skin prick testing with all concentrations diluted with normal saline did not trigger irritating reactions. Third, they used a 1:100,000 dilution (equivalent to a concentration of 1.25 µg/ml) for intradermal testing. Overall, the authors concluded that skin testing for omalizumab is safe and well tolerated (38).

Another group reported their skin testing experience in patients with hypersensitivity reactions to dupilumab. In this study, the authors determined that skin prick testing with undiluted dupilumab (150 mg/ml) and intradermal testing with concentrations up to 15 mg/ml were non-irritating in the two patients with atopic conditions and six controls (7). Interestingly, one of the patients who developed delayed angioedema and pruritus after dupilumab developed a delayed reaction to the intradermal testing. This result suggests skin testing might have diagnostic value beyond IgE-mediated hypersensitivity reactions.

Limitations of skin testing for monoclonal antibodies include lack of validation, unknown negative predictive value, and the cost of the medications. Furthermore, since adverse drug events to monoclonal antibodies include non-IgE-mediated reactions, such as complement activation and serum sickness-like reactions, clinicians much be cautious when determining if pursuing skin testing is the best option for their patients. Given these limitations, the recently published practice parameter updates on drug allergy state that skin testing for monoclonal antibodies should rarely be performed in a clinical setting (16).

## Desensitization protocols for biologics

Drug desensitization is defined as a procedure that allows for a temporary induction of drug tolerance. By performing such a procedure, an individual's drug response is modified, thereby allowing a drug that had previously caused a hypersensitivity reaction to be used safely on a temporary basis. Desensitization is typically performed to address IgE-mediated immediate reactions and is considered when the implicated drug is the preferred therapy. After a desensitization procedure, the achieved drug tolerance state is temporary and is maintained only if the specific medication is continually used (16). Drug desensitization procedures are not without risks and should only be performed by experienced personnel who have the ability to recognize and readily treat any reactions, including anaphylaxis, that may occur (40). Prior to recommending drug desensitization, switching to alternate medications with equal or similar efficacy should be evaluated (9). Desensitization is contraindicated if the incident event was a severe non–IgE-mediated reaction, such as Stevens-Johnson syndrome, toxic epidermal necrolysis, or exfoliative dermatitis (40).

When considering biologics used for allergic diseases, published data for desensitization procedures is limited to omalizumab (41–44), which does have a black box warning for risk of anaphylaxis. The largest cohort reported that underwent desensitization for omalizumab includes 12 patients, four of whom experienced reactions upon first exposure. The remaining eight had reactions within the first three exposures to omalizumab (41). Within this cohort, 67% had a Brown grade 2 initial reaction, and 33% had a Brown grade 3 initial reaction (45). Allergy skin testing was not performed during the allergy evaluation. The desensitization procedure consisted of a 7-step protocol that began with a 1:10 dilution of omalizumab with subsequent increasing doses administered by subcutaneous injection 30 min after the previous dose. If no reactions occurred during the initial desensitization procedure, the protocol steps were incrementally consolidated with eventual graduation to regular injection in clinic if the desensitization was tolerated. Premedication was administered 30 min prior to the first dose of the desensitization procedure. In total, the cohort underwent 97 omalizumab desensitizations, and 93 were completed with either no reaction or limited cutaneous symptoms. Six patients were able to return to routine clinic injections for continued omalizumab treatment (41).

Aside from omalizumab, data is severely lacking when considering desensitization procedures for other biologics used for allergic diseases. For this situation, current guidelines suggest desensitization protocols employed successfully for other monoclonal antibodies, such as rituximab, infliximab, and tocilizumab, that are used to treat non-allergic conditions (9, 16, 40). For intravenous medications, these desensitization protocols typically consist of 1–4 solutions of different drug dilutions that are administered in 4, 8, 12, or 16 steps with 2-–2.5-fold dose increments. A 7-step desensitization protocol similar to that described for omalizumab may be utilized for subcutaneous biologics. In this 7-step protocol, a 1:10 dilution of the original drug concentration is used for the first 4 steps with the original concentration being used for the remaining 3 steps. Doses are administered every 30 min with a doubling of dose with each step until the target dose is achieved. Premedications are typically administered for these monoclonal antibody desensitization protocols (17).

## Discussion

Among biologics used to treat allergic diseases, anaphylaxis has been reported to omalizumab, mepolizumab, dupilumab, reslizumab and benralizumab. Hypersensitivity reactions to omalizumab are the most well-characterized (4, 16). There are several unusual features of anaphylactic reactions to omalizumab, including a relatively high percentage of events occurring after the first dose and the potential for delayed anaphylactic reactions (32). Further characterization of immediate hypersensitivity reactions to omalizumab and other biologics would be useful (e.g., timing of symptom onset in relation to biologic administration, patient characteristics, etc.). Such information would aid in risk stratification for clinicians utilizing these medications. It may also shed light on the pathophysiology underlying anaphylaxis to biologic agents, which could facilitate greater accuracy in the diagnostic approach. For example, if excipient allergy is thought to be the cause of hypersensitivity reactions in a substantial number of patients, skin testing to relevant excipients may be a useful diagnostic tool. Limitations include low incidence of anaphylactic events overall, and the reliance on retrospective case reports and case series to describe these events.

Further characterization of serum sickness and type IV hypersensitivity reactions face similar challenges, with the added difficulty of greater diagnostic uncertainty as there is a lack of consensus definition for serum sickness reaction (9). There are no diagnostic tools available to determine the risk of reintroducing an agent to which a patient experienced a suspected SSR or SSLR. A few patients who developed SSLRs while participating in omalizumab clinical trials were able to continue therapy without interruption. Challenge could be considered for patients who develop SSLRs to omalizumab after shared decision making, though data is limited to a handful of patients (16).

Skin testing is currently the most useful tool available for risk stratification when deciding whether a patient with history of immediate allergic reaction may benefit from challenge or desensitization. Most of the available data regarding skin testing and desensitization is specific to omalizumab. Data regarding skin testing and desensitization for other biologics used for allergic diseases is severely lacking. Future studies should establish standardized non-irritating concentrations for anti-IL-5 monoclonal antibodies (mepolizumab, reslizumab, and benralizumab), tezepelumab, and tralokinumab. Validating these non-irritating concentrations will allow a more accurate interpretation of the skin testing results. Given the high cost of monoclonal antibodies for allergic diseases, there needs to be increased access to test solutions for skin testing. In this regard, partnerships with pharmaceutical companies producing these monoclonal antibodies might be worth exploring.

Further exploration regarding optimal approaches to diagnosis and management of hypersensitivity reactions to biologics is of high importance, as these medications can offer transformative disease control for many patients with allergic diseases. As such, the allergist and immunologist plays a crucial role in both diagnosing and managing hypersensitivity reactions to these agents.
